# Antioxidant, Larvicidal, and Cytotoxic Studies on *Asplenium aethiopicum* (Burm. f.) Becherer

**DOI:** 10.1155/2014/876170

**Published:** 2014-08-26

**Authors:** Johnson Marimuthu alias Antonysamy, Gowtham Janarthanan, Sivaraman Arumugam, Janakiraman Narayanan, Narayani Mani

**Affiliations:** Centre for Plant Biotechnology, Department of Botany, St. Xavier's College (Autonomous), Palayamkottai, Tamil Nadu 627 002, India

## Abstract

The present study was intended to determine the antioxidant, larvicidal, and cytotoxic potential of various extracts of *Asplenium aethiopicum* (Burm. f.) Becherer. Antioxidant potential of the extracts was determined by the DPPH radical scavenging, phosphomolybdenum, and scavenging of H_2_O_2_. Larvicidal activity of *Asplenium aethiopicum* was performed against the fourth instar larvae *Culex quinquefasciatus*. Cytotoxic activity was analysed in terms of brine shrimp lethality bioassay. The best free radical scavenging activity was exerted by methanolic extract of *Asplenium aethiopicum* (IC_50_ 91.4 *μ*g/mL) followed by acetone extract (IC_50_ 99.8 *μ*g/mL). Highest larval mortality was observed in the crude acetone extracts of *Asplenium aethiopicum* against *Culex quinquefasciatus* (LC_50_ = 166.6 ppm) followed by methanolic extracts. Acetone extract of *Asplenium aethiopicum* was found to be most effective at which 50% and 90% mortality of brine shrimp nauplii that occurred were found to be 192.8 and 434.3 ppm, respectively. The results of the present study revealed the antioxidant, larvicidal, and cytotoxic potential of *Asplenium aethiopicum*.

## 1. Introduction

Free radicals which have one or more unpaired electrons (superoxide, hydroxyl, and peroxyl) are produced in normal or pathological cell metabolism and the compounds that can scavenge free radicals have great potential in ameliorating the diseases and pathological cells [1]. Antioxidants play an important role to protect the human body against damage by reactive oxygen species. Plants containing bioactive compounds have been reported to possess strong antioxidant properties [2]. Screening of various bioactive compounds from plants has led to the discovery of new medicinal drugs which have efficient protection and treatment roles against various diseases [3–5].

Mosquito-borne diseases are major threat to over 2 billion people in the tropics. Several mosquito species belonging to genera* Culex, Anopheles,* and* Aedes* are vectors for the pathogens of various diseases like malaria, filariasis, Japanese encephalitis, dengue, yellow fever, and chikungunya [6]. The major problems associated with the use of chemicals for the control of pests including mosquitoes include the development of resistance to the chemicals, issues around the residues in animals and the environment, and their undesirable side effects [7]. Extracts from plants may be alternative sources of mosquito control agents, since they constitute a rich source of bioactive compounds that are biodegradable into nontoxic products and potentially suitable for use to control mosquitoes.

*Artemia salina *L. (Artemiidae), the brine shrimp, is an invertebrate of the fauna of saline aquatic and marine ecosystems. It can be used in laboratory bioassay in order to determine toxicity through the estimation of medium lethal concentration (LC_50_ values) which has been reported for series of toxins and plant extracts. Several naturally extracted products which had LC_50_ < 1000 *μ*g/mL using brine shrimp lethality bioassay (BSLB) were known to contain physiologically active principles [8]. BSLB and other* in vivo *lethality tests have been successively employed for bioassay guide fractionation of active cytotoxic and antitumor agents [9, 10].

Plant extracts especially phytochemicals (plant derived chemicals) in general have been recognized as an important natural resource and can play an important role against various infectious diseases at the individual as well as at the community level [11, 12]. Pteridophytes are an important component of the flora of our major region of species diversity, next to angiosperms in number. Different species of ferns have been subjected to pharmacological studies to know the mechanism of action and to know the application of these drugs for the welfare of human beings without any other side effects. India, being a vast country rich in pteridophytes, will be more practical if detailed studies are undertaken on this group. Hence, the present study was intended to study the antioxidant, larvicidal, and cytotoxic potential of* Asplenium aethiopicum *(Burm. f.) Becherer.

## 2. Materials and Methods

### 2.1. Collection of Plant Material

Healthy, disease-free ferns of* Asplenium aethiopicum *(Burm. f.) Becherer were collected near Venkatraman Bridge, Ooty-Gudalur road, Nilgiris, Tamil Nadu, India. The collected samples were brought to the laboratory in plastic bags. They were washed with tap water followed by distilled water to remove the unwanted debris. The plant was blotted on the blotting paper and spread out at room temperature in shade for 20 days. The shade dried samples were ground to fine powder using tissue blender. The powdered samples were then stored in refrigerator at 4°C for further analysis.

### 2.2. Preparation of Extracts

The dried and powered plant materials (30 g) were successively extracted with 180 mL of petroleum ether, chloroform, acetone, and methanol by using Soxhlet extractor for 8 h at a temperature not exceeding the boiling point of the solvent. After evaporation of the solvent under reduced pressure, the weight of each extract was 25.5 g/30 g (85%) for the methanolic extract, 18 g/30 g (60%) for acetone, 17.4 g/30 g (58%) for chloroform, and 6.3 g/30 g (21%) for the petroleum extract.

### 2.3. Preliminary Phytochemical Analysis

The different extracts were tested for steroids, terpenoids, alkaloids, phenolic compounds, saponins, tannins, flavonoids, cardiac glycosides, sterols, and amino acids according to the method described by Harborne [13].

### 2.4. Determination of Total Phenolics and Flavonoids

The total phenolic content was determined according to the method described by Siddhuraju and Becker [14]. The flavonoid contents of all the extracts were quantified as it acts as a major antioxidant in plants, reducing oxidative stress, estimated as described by Zhishen et al. [15].

### 2.5. Antioxidant Activity

#### 2.5.1. DPPH Radical Scavenging Activity

The antioxidant activity of different extracts was determined in terms of hydrogen donating of radical scavenging ability using the stable radical DPPH, according to the method of Brand-Williams et al. [16].

#### 2.5.2. Phosphomolybdenum Assay

The total antioxidant activity was evaluated by the green phosphomolybdenum complex formation according to the method described by Prieto et al. [17]. The results are mean values expressed as g of ascorbic acid (AA) equivalents/100 g extract.

#### 2.5.3. Scavenging of Hydrogen Peroxide

The ability of extracts to scavenge hydrogen peroxide was determined according to the method of Ruch et al. [18]. The percentage inhibition activity was calculated using the formula

(1) %  scavenging  activity  =[(Control  OD−Sample  OD)Control  OD]×100.

### 2.6. Larvicidal Activity

*Culex quinquefasciatus *(IV instar) larvae were collected from and around Tirunelveli district (sewage) with the help of “O” type brush. These larvae were brought to the laboratory and transferred to 18 × 13 × 4 cm size enamel trays containing 500 mL of water maintained in the laboratory.* C. quinquefasciatus *was maintained at 27 ± 2°C, 75–85% RH and 14L: 10D photoperiod cycles. Fourth instar larvae of* C. quinquefasciatus *were transferred in 250 mL glass beaker containing desired plant extracts (petroleum ether, chloroform, acetone, and methanol) concentration such as 50, 100, 150, 200, and 250 ppm. The plant extracts were dissolved with acetone for the larvicidal activity. Each and every experiment was performed with five replicates and repeated thrice. 1% of acetone was used as negative control. The control mortality was corrected by Abbott's formula and LC_50_ and LC_90_ regression equation and 95% confidence limits of lower (LCL) and upper confidence limits (UCL) were calculated by using probit analysis. The standard larvicide Temephos (Abate) was used as positive control.

### 2.7. Cytotoxic Activity

Artificial sea water (38 g NaCl/1000 mL tap water) was taken in small tank and shrimp eggs were added to one side of the divided tank and the side was covered. The shrimps were allowed for 48 hrs to hatch and mature as nauplii. The hatched shrimps were taken for bioassay. Dried methanolic extract of* A. aethiopicum* was taken in different concentrations (2.5, 5, 7.5, 10, and 12.5 mg/10 mL) to the sample tubes. The methanolic extracts were dissolved with DMSO for the cytotoxic activity. With the help of a Pasteur pipette 10 living shrimps were dropped into each test tube. Control group was added in cytotoxic activity to validate the test method and result obtained due to the cytotoxic activity of the test agent. After 24 hours, the tubes were inspected using a magnifying glass and the number of survived nauplii in each vial was counted and observations were recorded for each vial. Each and every experiment was performed with five replicates and repeated thrice. Using the recorded observations LC_50_, 95% confidence limit, LC_90_, and chi square values were calculated. The standard plumbagin was used as positive control.

## 3. Results and Discussion

### 3.1. Phytochemical Analysis

The results of preliminary phytochemical analysis of* A. aethiopicum* were demonstrated in Table 1. Methanol, chloroform, acetone, and aqueous extracts of* A. aethiopicum* showed the maximum presence of five metabolites (Table 1).

### 3.2. Determination of Total Phenolics and Flavonoids

The total phenolics content of* A. aethiopicum *petroleum ether, acetone, chloroform, and methanolic extracts was determined and presented in Table 2. Maximum amount of extractable total phenolics (453.4 mg GAE/g) was recorded in acetone extracts of* A. aethiopicum *followed by methanolic extracts. Chloroform extracts of* A. aethiopicum *displayed the lowest level of total phenolics (44.6 mg GAE/g) presence. The total flavonoid contents of petroleum ether, acetone, chloroform, and methanolic extracts of* A. aethiopicum* varied considerably from 156.6 to 358.8 mg GAE/g (Table 2). Highest amount of flavonoids (358.8 mg GAE/g) was present in acetone extracts followed by methanolic and chloroform extracts of* A. aethiopicum,* respectively. Quantitative estimation of phenolics and flavonoids in various extracts of* A. aethiopicum *determined the presence of extractable compounds with varied percentage.

Phenols and phenolic compounds are greatly used in skin infections, wound healing, inflammation, antioxidant, immune enhancers, anticlotting, and hormone modulators [19]. Flavonoids have a membrane permeability effect and are considered as potential antioxidants and have protective action against allergies, inflammation, free radical, platelet aggregation, microbes, ulcers, hepatotoxins, viruses, and tumor [20, 21]. The present study results of* A. aethiopicum* showed the presence of phenolic compounds and flavonoids which are useful as natural antioxidants.

### 3.3. Antioxidant Activity

The free radical scavenging activity of* A. aethiopicum* petroleum ether, acetone, chloroform, and methanolic extracts was depicted in Table 3. The best free radical scavenging activity was exerted by methanolic extract of* A. aethiopicum* (IC_50_ 91.4 *μ*g/mL) followed by acetone extract (IC_50_ 99.8 *μ*g/mL). Chloroform extracts of* A. aethiopicum* showed comparable levels of free radical scavenging activity (IC_50_ 171.4 *μ*g/mL). The free radical scavenging activity was found to be the least in petroleum ether extracts of* A. aethiopicum*.

The results of phosphomolybdenum assay of petroleum ether, acetone, chloroform, and methanolic extracts of* A. aethiopicum* were illustrated in Table 3. Among the various extracts tested, methanolic extracts of* A. aethiopicum* had the strongest phosphomolybdenum reduction (188.9 g AA/100 g). The remaining three extracts of* A. aethiopicum* showed lower level of phosphomolybdenum reduction. The petroleum ether, acetone, chloroform, and methanolic extracts of* A. aethiopicum* showed an efficient H_2_O_2_ scavenging activity in concentration-dependent manner (Table 3). Methanolic extracts of* A. aethiopicum* showed maximum inhibition (93.8%) followed by acetone and chloroform extracts (83.5 and 73.9%), respectively. Hydrogen/electron transfer from antioxidants to DPPH radical and Mo (VI) complex occur in the DPPH radical and phosphomolybdenum assays, respectively [22]. This reduction ability was relatively shown by the active extracts of* A. aethiopicum*. The results of H_2_O_2_ scavenging activity seem to be an effective scavenger of nitric oxide H_2_O_2_. H_2_O_2_ can be converted to more reactive species (e.g., the hydroxyl radical), which has been thought to have one of the unfavourable effects caused by it. Hence, the results indicated that* A. aethiopicum *can act as a good scavenger of such harmful radicals.

### 3.4. Larvicidal Activity

All the four extracts of* A. aethiopicum* showed moderate larvicidal effect after 24 hrs (Table 4). However the highest larval mortality in terms of lethal concentrations for 50% mortality was observed in the crude acetone extracts of* A. aethiopicum* against* C. quinquefasciatus* (LC_50_ = 166.6 ppm) followed by methanolic extracts (LC_50_ = 181.2 ppm). Chloroform extract of* A. aethiopicum* appeared to have lowest effect against* C. quinquefasciatus* (LC_50_ = 288.9 ppm) when compared to other extracts. The positive control Temephos showed 100% of mortality at 0.025 ppm.

Many approaches have been developed to control mosquito menace. One such approach to prevent mosquito-borne disease is by killing mosquito at larval stage. The current mosquito control approach is based on synthetic insecticides. Even though they are effective, they created many problems like insecticide resistance [23], pollution, and toxic side effect on human beings [24]. Therefore, it is necessary to look for and find the better larvicide which could provide a safer and long-lasting control against all mosquito species. Plant extracts, especially botanical larvicides, provide an alternative to synthetic insecticide because they are generally considered safe, are biodegradable, and can often be obtained from local sources. In addition, the use of medicinal plants for mosquito control is likely to generate local employment, reduce dependence on expensive imported products, and stimulate efforts to enhance public health. In the present study, larvicidal effects of petroleum ether, chloroform, acetone, and methanolic extracts of* A. aethiopicum *against the fourth instar larvae* C. quinquefasciatus* were tested and the highest larval mortality in terms of lethal concentrations for 50% mortality was observed in the crude acetone extracts which could be helpful to be applied in integrated control strategies to gain maximum impact on vector control.

### 3.5. Cytotoxic Activity

The petroleum ether, acetone, chloroform, and methanolic extracts of* A. aethiopicum* showed different mortality rate of brine shrimp which increased proportionally with the increasing concentration of the extract. The inhibitory effect of the extract might be due to the toxic compounds present in the crude extracts. The acetone extract of* A. aethiopicum* was found to be most effective at which 50% mortality (LC_50_) and 90% mortality (LC_90_) of brine shrimp nauplii that occurred were found to be 192.8 and 434.3 ppm, respectively (Table 5). Chloroform extract showed less cytotoxicity (LC_50_ = 353.2 ppm) than other fractions. The 0.046 ppm of standard plumbagin showed 100% mortality of brine shrimp nauplii.

Bioactive compounds are almost always toxic in high doses. Thus* in vivo* lethality in a simple zoologic organism can be used as a convenient monitor for screening and fractionation in the discovery of and monitoring bioactive natural compounds [25]. The brine shrimp lethality test is considered to be very useful in determining various biological activities such as cytotoxic, phototoxic, pesticidal, trypanocidal, and enzyme inhibition and ion regulation activities [26]. Recently, there has been interest in the brine shrimp lethality assay as a means of detecting ion regulation or ion-channel activity such as that involving Na^+^-K^+^-ATPase or calcium channels [27, 28]. It can also be extrapolated for cell-line toxicity and antitumor activity [29]. This is a rapid method utilizing only 24 hours, inexpensive, and needs no special equipment. It is so simple that no aseptic technique is required. It utilizes a large number of organisms for validation and relatively few samples. It does not require animal serum as needed for other methods of cytotoxicity testing [30]. This bioassay has been employed to determine cytotoxic activity of several extracts. In the present study, the different extracts of* A. aethiopicum *showed a marked dose-dependent cytotoxic activity in terms of brine shrimp lethal effect. The lethality of acetone extract of* A. aethiopicum *was found to be most effective which might be due to the toxic compounds present in the crude extracts.

## 4. Conclusion 

In the current study, it can be confirmed that the extracts of* A. aethiopicum *defend against harmful free radicals and can find use as broad-spectrum antioxidative agent after extensive investigation. Larvicidal effects of* A. aethiopicum *could be helpful to apply in integrated control strategies to gain maximum impact on vector control. Cytotoxic activity of* A. aethiopicum *in terms of brine shrimp lethal effectwas found to be most effective for isolating biogenic compounds from plant extracts.

## Figures and Tables

**Table 1:** Preliminary phytochemical analysis on *A. aethiopicum. *

Compounds	Pet. ether	Chloroform	Acetone	Methanol	Aqueous
Steroids	−	+	+	+	−
Alkaloids	−	−	−	+	−
Phenolic groups	+	+	+	+	+
Cardiac glycosides	−	+	+	+	+
Flavonoids	+	+	+	+	+
Saponins	−	−	−	−	+
Tannins	−	−	−	−	+
Amino acids	−	−	−	−	−
Terpenoids	−	−	−	−	−
Sterols	−	+	+	−	−

**Table 2:** Total phenolics and flavonoids contents of *A. aethiopicum. *

Extracts	Phenolics (mg GAE/g ± SD)	Flavonoids (mg GAE/g ± SD)
Methanol	371.4 ± 2.3	261.1 ± 15.2
Chloroform	44.6 ± 3.2	233.3 ± 15.2
Pet. ether	89.4 ± 7.1	156.6 ± 17.6
Acetone	453.4 ± 25.4	358.8 ± 13.8

**Table 3:** DPPH radical scavenging activity, phosphomolybdenum assay, and hydrogen peroxide scavenging activity of various extracts of *A. aethiopicum. *

Extracts	DPPH IC_50_ (*μ*g/mL)	Phosphomolybdenum assay (g AA/100 g extract)	Hydrogen peroxide scavenging activity
Methanol	91.4	188.9 ± 4.8	93.8 ± 0.5
Chloroform	171.1	20.2 ± 1.2	73.9 ± 1.9
Pet. ether	514.4	22.9 ± 0.2	67.4 ± 0.7
Acetone	99.8	26.2 ± 0.4	83.5 ± 2.8

**Table 4:** Larvicidal activity of *A. aethiopicum* against *C. quinquefasciatus. *

Extracts	LC_50_ (ppm)	LCL	UCL	LC_90_ (ppm)	*χ* ^2^ (df)
Pet. ether	268.1	242.2	292.3	524.2	2.712
Chloroform	288.9	261.7	315.6	357.8	0.412
Acetone	166.6	62.1	224.6	290.4	9.216
Methanol	181.2	123.8	222.9	518.2	0.846

LC_50_ = lethal concentration for 50% larval mortality; LC_90_ = lethal concentration for 90% larval mortality;LCL = lower confidence limit; UCL = upper confidence limit.

**Table 5:** Cytotoxicity of *A. aethiopicum* against *A. salina. *

Extracts	LC_50_ (ppm)	LCL	UCL	LC_90_ (ppm)	*χ* ^2^ (df)
Pet. ether	302.2	269.8	335.8	655.1	3.512
Chloroform	353.2	318.6	394.6	745.1	1.791
Acetone	192.8	162.9	217.8	434.3	1.823
Methanol	342.6	307.6	383.3	737.8	1.419
